# Supplementary figures and images for: Estrogen impacts NOD2-dependent regulation of intestinal homeostasis
Source: PLoS Biol. 2026 Jun 9;24(6):e3003766. doi: 10.1371/journal.pbio.3003766 (PMC13271492; doi:10.1371/journal.pbio.3003766)

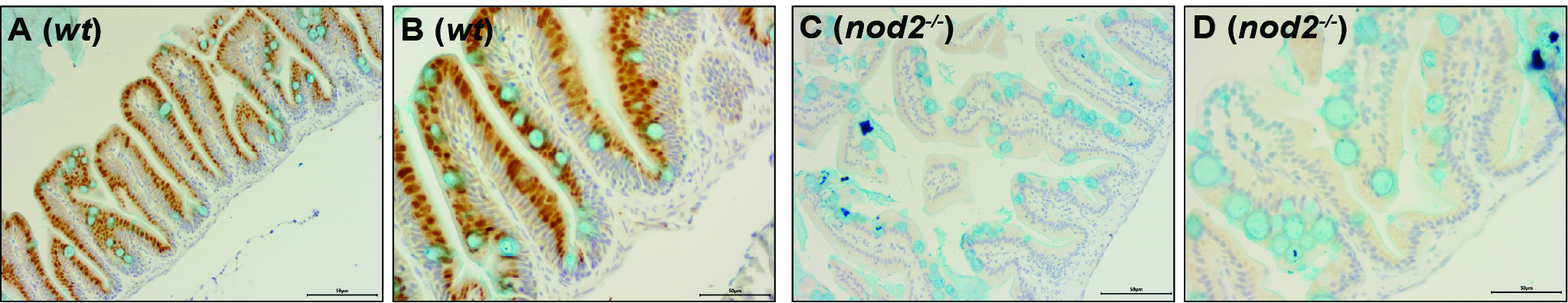

Supplement: S1 Fig — A–D) Representative sagittal sections of 4-month-old cohoused WT and nod2−/− adult intestines immunostained with a custom anti-zebrafish NOD2 antibody. Images were taken at 20× (A, C) and 40× (B, D) magnification. n = 5 fish per genotype (mixed sex). Scale bars = 50 μm. Icon indicates the developmental stage at which the experiment was performed. (TIF) [file pbio.3003766.s001.tif]

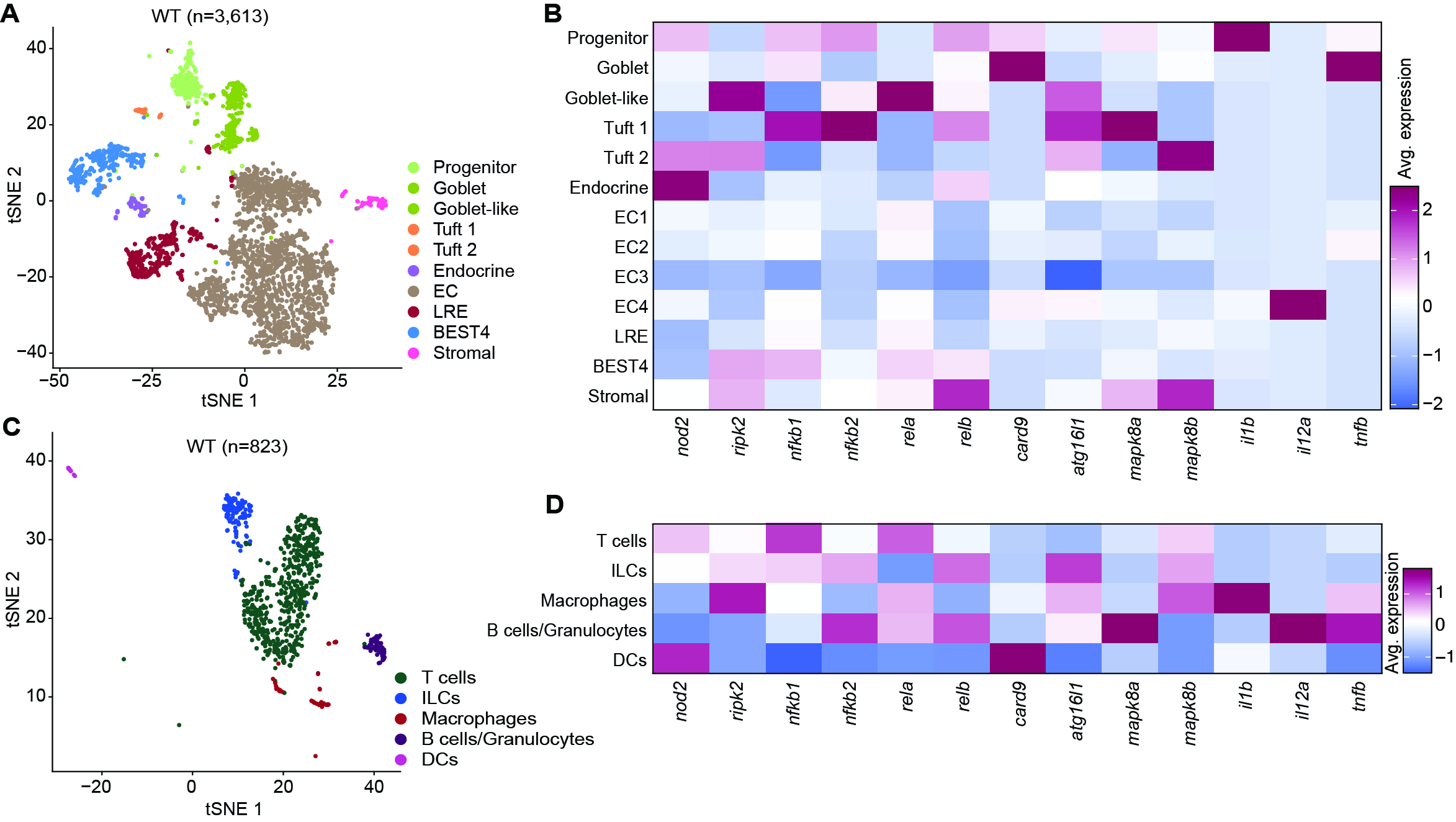

Supplement: S2 Fig — A, B) tSNE plot (left) of single-cell RNA-seq from WT adult epithelial and stromal cells (n = 3,613), annotated by cell type. Heatmap (right) shows average expression of nod2 and NOD2-pathway genes across epithelial and stromal clusters. C, D) tSNE plot (left) of single-cell RNA-seq from WT adult intestinal immune cells (n = 823), annotated by lineage. Heatmap (right) shows average expression of nod2 and pathway-associated genes across immune subsets. The data underlying the graph in this figure are available in S11 Data. (TIF) [file pbio.3003766.s002.tif]

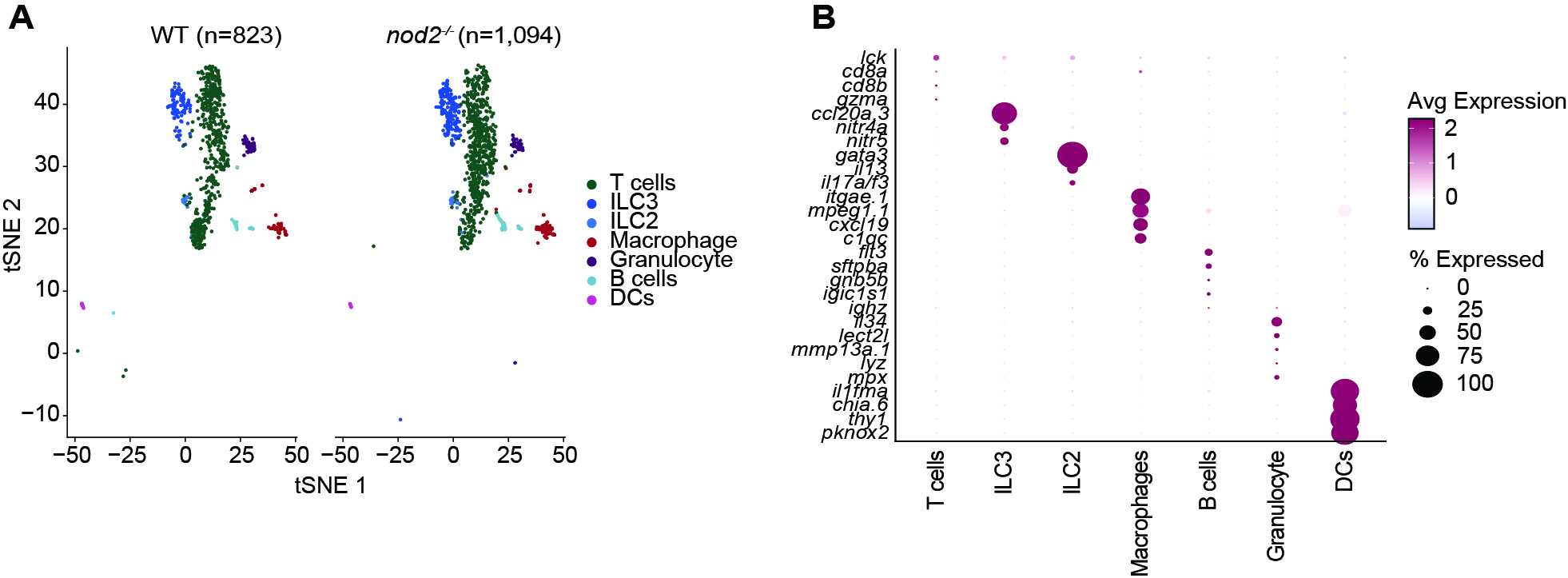

Supplement: S3 Fig — A) tSNE visualization of leukocytes from WT (left) and nod2−/− (right) zebrafish intestines (6-month-old, cohoused), colored by immune cell type based on transcriptional clustering and marker gene expression. Major immune subsets identified include T cells, ILC3s, ILC2s, macrophages, granulocytes, B cells, and dendritic cells (DCs). B) Dotplot representation of the expression levels of established markers in the indicated cell types from integrated data set. The data underlying the graph in this figure are available in S12 Data. (TIF) [file pbio.3003766.s003.tif]

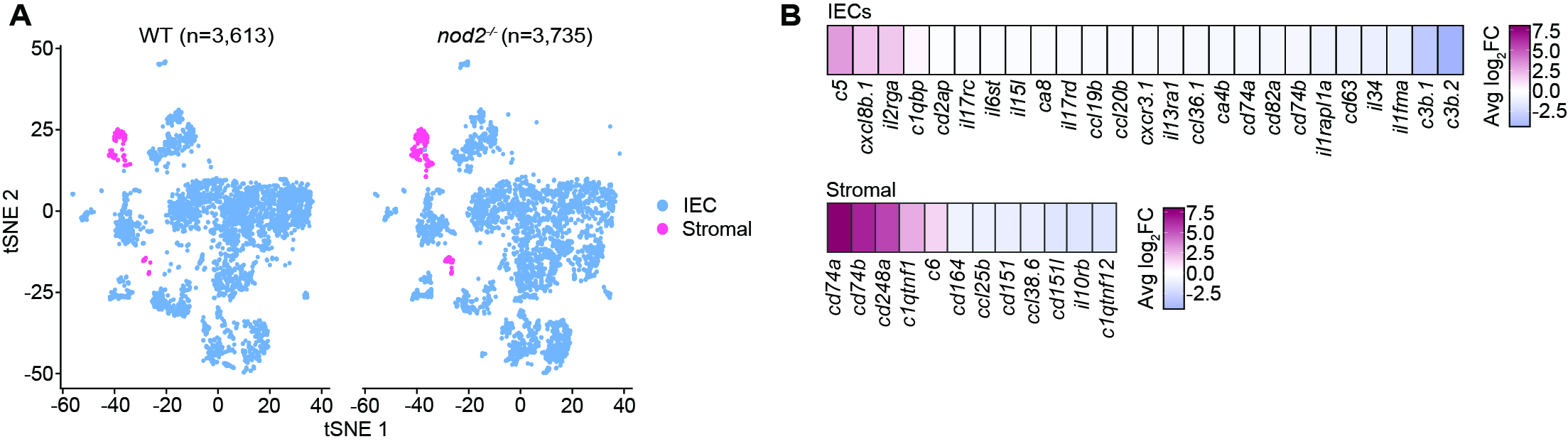

Supplement: S4 Fig — A) tSNE plots of intestinal epithelial cells (IECs; blue) and stromal cells (pink) from 6-month-old, cohoused WT and nod2−/− zebrafish. B) Heatmaps show differential expression of immune cell recruitment-associated genes in IECs and stromal cells. (For all genes adjusted P-value ≤ 0.05). The data underlying the graph in this figure are available in S13 Data. (TIF) [file pbio.3003766.s004.tif]

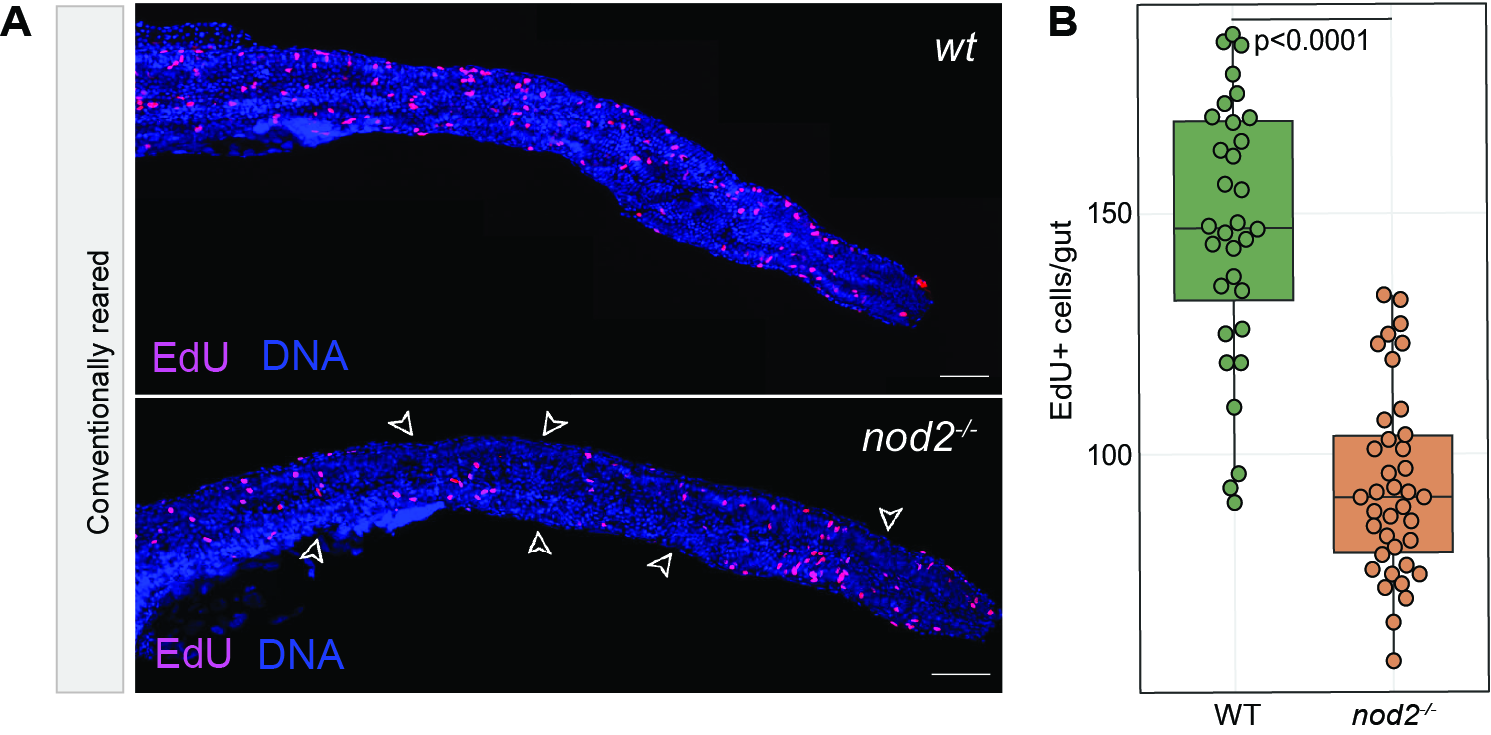

Supplement: S5 Fig — A) Immunofluorescence images of whole-mount intestines from conventionally reared WT and nod2−/− larvae (7 dpf), shown in rostral-to-caudal orientation from the base of the intestinal bulb to the cloaca. EdU⁺ cells (magenta) and nuclei (blue, DNA) are shown in merged false-colored images. White arrowheads highlight regions where nod2−/− intestines lacking EdU⁺ cells. Scale bars = 150 μm. B) Quantification of total EdU⁺ cells per gut in WT and nod2−/− larvae (7 dpf), determined from whole-mount confocal images by counting EdU⁺ nuclei across the entire intestinal epithelium. Each point represents an individual larva; nod2−/− intestines exhibit a significant reduction in proliferating cells. P-values were calculated using Mann–Whitney U test. Icon indicates the developmental stage at which the experiment was performed. The data underlying the graph in this figure are available in S14 Data. (TIF) [file pbio.3003766.s005.tif]

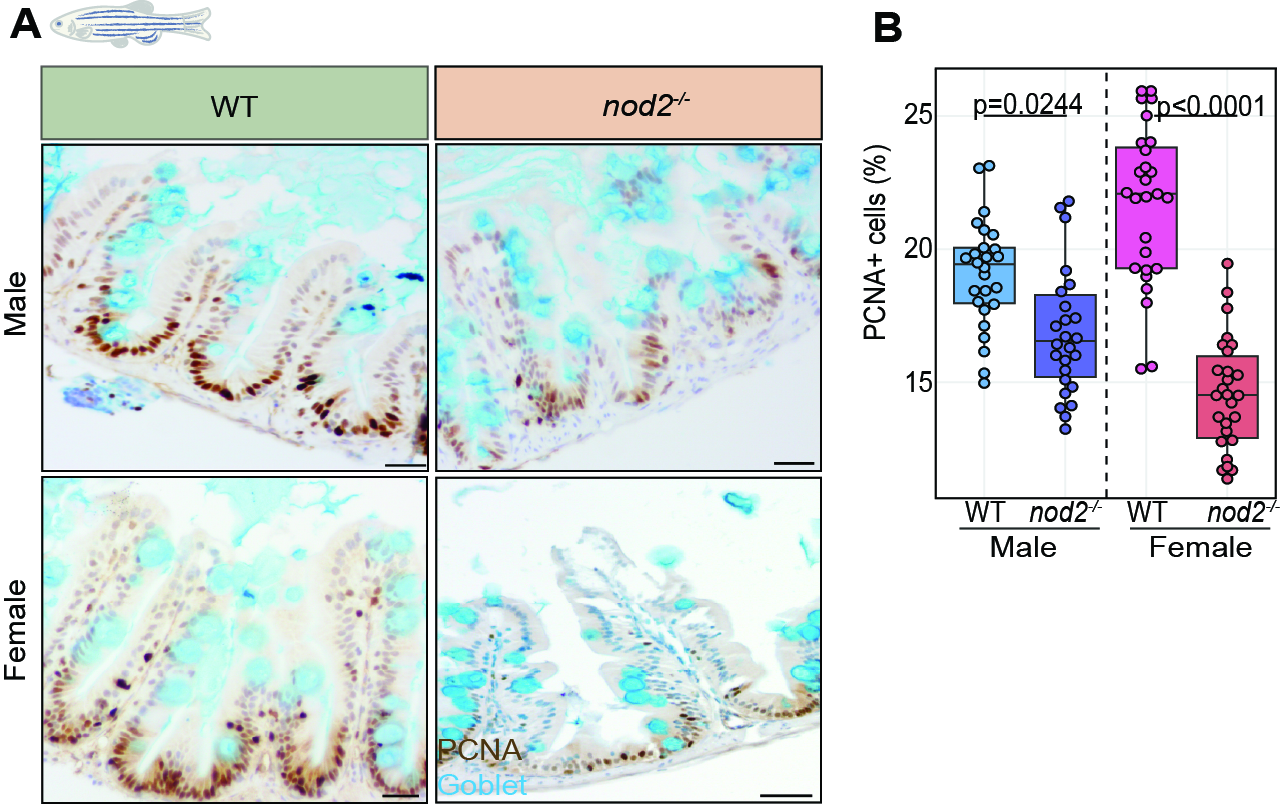

Supplement: S6 Fig — A) Representative sagittal sections of posterior intestine from 6-month-old WT and nod2−/− zebrafish, separated by sex. Sections were stained for proliferating cell nuclear antigen (PCNA; brown) and counterstained with Alcian blue to visualize goblet cells (cyan). Images are shown in rostral-to-caudal orientation and were collected from comparable posterior intestinal regions using identical imaging settings. Scale bars = 20 μm. B) Quantification of epithelial proliferation shown as the percentage of PCNA⁺ epithelial cells per villus in WT and nod2−/− intestines, analyzed separately in males and females. Multiple regions of interest were analyzed per fish; measurements were averaged per fish, and each point represents an individual measurement obtained from multiple images per fish intestine (n = 5 zebrafish per genotype). P-values were calculated using Mann–Whitney U test or Kruskal–Wallis test. Icon indicates the developmental stage at which the experiment was performed. The data underlying the graph in this figure are available in S15 Data. (TIF) [file pbio.3003766.s006.tif]

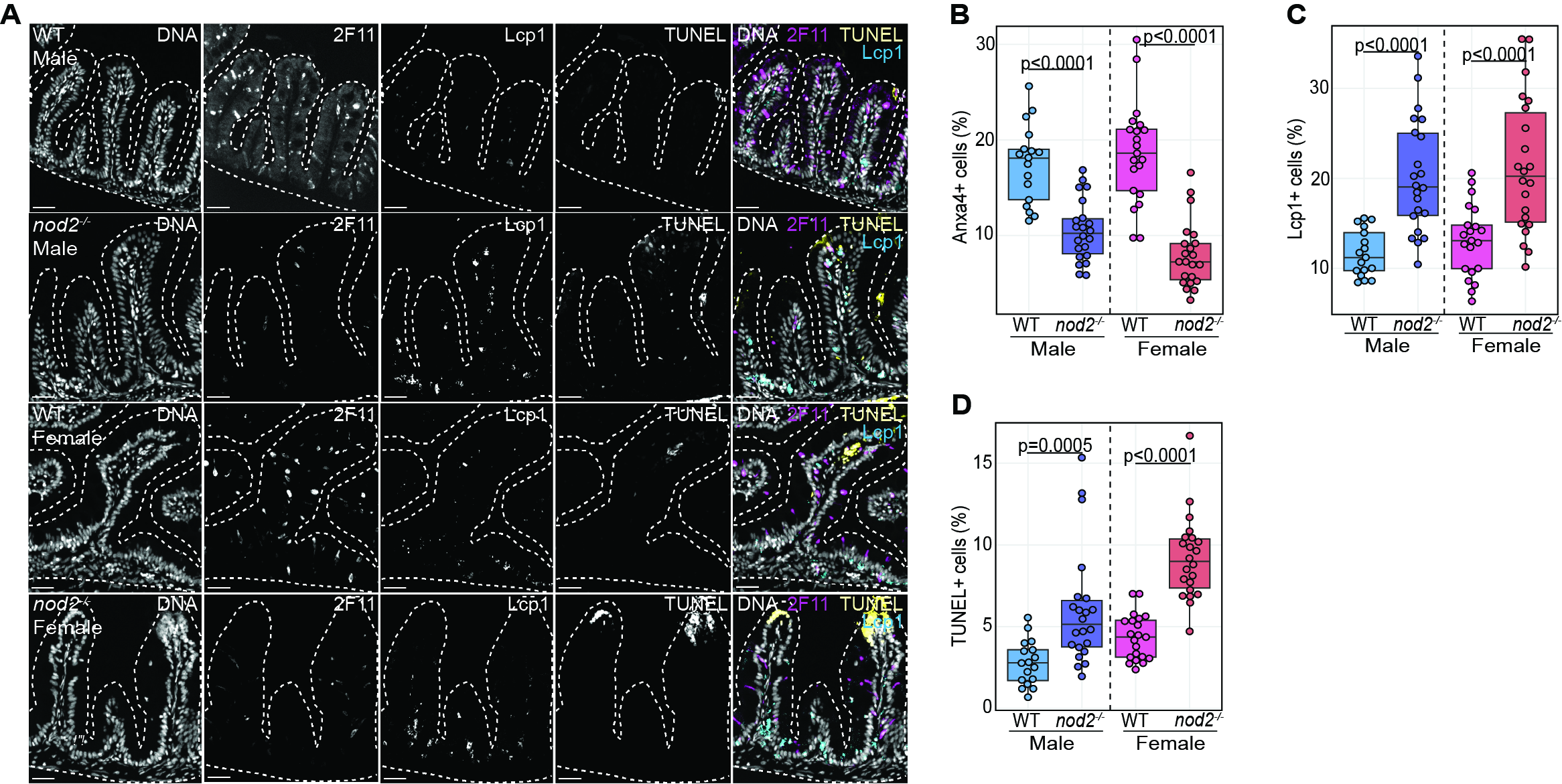

Supplement: S7 Fig — A) Representative sagittal sections of WT and nod2−/− intestines (6-month-old, cohoused adults) stained for DNA (nuclei), secretory cells (2F11/Anxa4), pan-leukocyte marker (Lcp1), and apoptotic cells (TUNEL). Merged panels show combined staining with outlined villus structures. Images are shown in rostral-to-caudal orientation. B–D) Quantification of % Anxa4+ cells (B), % Lcp1+ cells (C), and % TUNEL+ cells (D) from multiple sections of WT and nod2−/− intestines. Multiple regions of interest were analyzed per fish; measurements were averaged per fish, and each point represents an individual measurement obtained from multiple images per fish intestine (n = 5 zebrafish per genotype). Scale bars = 50 μm. P-values were calculated using Mann–Whitney U test or Kruskal–Wallis test. Icon indicates the developmental stage at which the experiment was performed. The data underlying the graphs in this figure are available in S16 Data. (TIF) [file pbio.3003766.s007.tif]

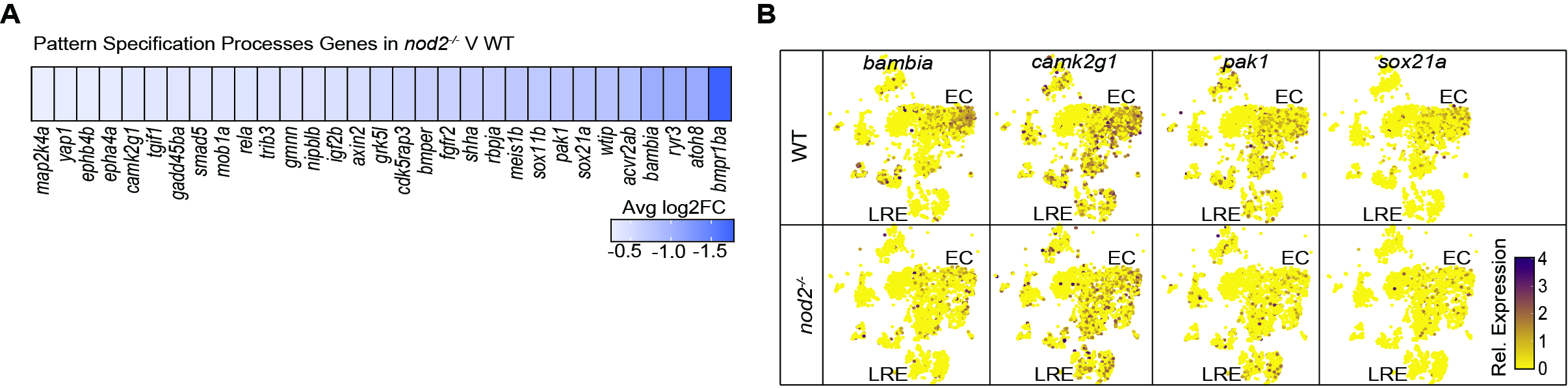

Supplement: S8 Fig — A) Heatmap illustration of representative pattern specification processes gene expression in nod2−/− versus WT adult intestinal epithelium (for all genes, adjusted p-value ≤ 0.05). B) Feature plots showing relative expression of select patterning genes (bambia, camk2g1, pak1, sox21a) in WT and nod2−/− intestinal epithelium. Expression is reduced across enterocyte (EC) and lower right epithelial (LRE) clusters in nod2−/− intestines, indicating broad loss of epithelial patterning cues. The data underlying the graphs in this figure are available in S17 Data. (TIF) [file pbio.3003766.s008.tif]
