# Supplementary material for: Estrogen impacts NOD2-dependent regulation of intestinal homeostasis
Source: PLoS Biol. 2026 Jun 9;24(6):e3003766. doi: 10.1371/journal.pbio.3003766 (PMC13271492; doi:10.1371/journal.pbio.3003766)
Supplement: Attachment — Submitted filename: 26_NOD2_Response_To_Reviewers.pdf [file pbio.3003766.s026.pdf]

Department of
Life Sciences

UNIVERSITY OF BATH

Bath BA2 7AY • United Kingdom
Prof Edan A. Foley
GSK Chair in Biomedical Microbiomics
Email ef796@bath.ac.uk

Dear Editors of PLoS Biology,

We would like to take this opportunity to thank you for organizing fair and comprehensive reviews of our article Estrogen Impacts NOD2-Dependent Regulation of Intestinal Homeostasis. We are grateful to all reviewers for their suggestions and believe that they have helped us to improve the quality of our initial submission.
In our revised manuscript, we provide additional data and improved text clarity to address all concerns and comments raised in the initial round of reviews. We hope that this revised manuscript is suitable for publication in PLoS Biology and are more than happy to answer any additional questions you may have. Below, we present all reviewer comments in bold, followed by our replies.

Sincerely,

Edan A. Foley

Reviewers' comments
Rev. 1: Stefan Oehlers – please note that this reviewer has signed the review.
Eklund & Foley use a novel zebrafish nod2 mutant allele to investigate the role of Nod2 in intestinal homeostasis, response to injury, and interaction with estrogen signalling. The manuscript's most novel finding is the interaction with estrogen signalling whereby Nod2 and E2/tamoxifen-regulated signalling appear to oppose each other. The discussion and limitation sections are quite clear about additional mechanistic experiments that *could* be performed to strengthen the manuscript's argument (such as use of genetics to target ER, additional small molecule probes). I am happy that sufficient experimental evidence is provided, most importantly in the form of a genetic tool combined with opposing small molecule probes with well characterised biological activity) to support the claims made in the manuscript and my comments are limited to deeper analysis of existing datasets and clarification points.
We appreciate this supportive assessment of our work by reviewer one and have worked through the text carefully to address all issues raised in their review.

Specific comments
Some of the histological analyses would benefit from deeper analyses. The regional effects, while not essential to the narrative of the Nod2 story, are useful to advance the comparative intestinal biology field.

Figure 1BC: there is an intestinal fold height difference in the later figures (Fig 3I, S1. Maybe not so strong in 2IJ) that should be described early in the story here.
We thank the reviewer for this helpful suggestion and agree that the intestinal architectural phenotype should be introduced earlier for clarity. We have revised the Results section describing

the generation and baseline characterization of the *nod2* mutant line to explicitly note that histological analysis revealed reduced epithelial fold height and altered mucosal organization in *nod2*$^{-/-}$ intestines, with reference to Supplementary Fig S1. We also ensured that later figures that quantify or illustrate intestinal structure (including Fig 3I and related panels) are cross-referenced accordingly. This change integrates the fold-height phenotype into the early narrative and improves continuity across the Results section.

Figure 2K could be quantified to comment on the apical vs basal/LP location of leukocytes. From the example images it appears the increase in leukocyte density is driven by leukocytes at the base of the intestinal folds. It might be difficult to prove, but this would correlate with stroma>IEC expression of chemoattractant/adhesion genes in NOD2-/-.
We agree that the spatial distribution of leukocytes along the intestinal fold axis is an important point. In response to the reviewer's suggestion, we have now extended our analysis to include positional stratification of Lcp1^{+} cells along the fold axis. Using the existing stained sections and images, we subdivided the mucosa into anatomical compartments (villus tip, mid-fold length, basal region, and stromal/lamina propria compartment) and quantified the percentage distribution of Lcp1^{+} cells across these zones. This new spatial quantification has been added as Fig. 2L and is described in the Results section. This analysis shows that the increase in leukocytes in *nod2*$^{-/-}$ intestines is not restricted to a single apical or basal compartment but instead reflects a broader expansion across epithelial and stromal regions. This pattern is consistent with our single-cell transcriptomic data showing altered chemokine and adhesion gene expression across multiple intestinal cell types, including stromal and epithelial populations. We have updated the figure legend and Results text to include the new analysis.

Fig S5 and 3F: please be more clear about the location (along the intestinal bulb-cloaca axis) of tissue shown as examples and enumerated. I think the right edge of the S5 images are the cloaca but the framing of the 3F images is not immediately obvious.
We concur that the study would benefit from precise description of anatomical position in each figure. We have revised the figure legends to explicitly state the anatomical orientation and intestinal region analysed in all whole-mount intestine images and quantifications. For Figs 3F and S5, we now indicate that images are displayed in rostral-to-caudal orientation from the base of the intestinal bulb to the cloaca, and we specify the intestinal segment or region of interest used for quantification. These clarifications have been added consistently across all relevant figure legends to avoid ambiguity in tissue location and orientation.

Figure 3H: Can the authors explain why they switched to a flow cytometry methodology here? The whole mount imaging approach would allow detection of spatial patterns to NOD2/MDP-dependent proliferation that are missed by the analysis of dissociated cells.
We thank the reviewer for highlighting this point and agree that the original text did not sufficiently explain the rationale and implementation of the different proliferation assays. We have revised the Results, Methods, and figure legends to clarify both the methodology and the reporting metrics. Imaging-based EdU quantification was performed using whole-mount confocal microscopy, with EdU^{+} nuclei counted either across the entire intestine (baseline genotype comparisons; Supplementary Fig. 5) or within a standardized intestinal region of interest (ROI) (rearing-condition comparisons; Fig. 3G). In ROI-based analyses, EdU^{+} cells are reported as a percentage of total epithelial nuclei in the quantified region. We now explicitly state the quantified region, denominator, and counting approach in both the Methods and figure legends. Flow cytometry (Fig. 3H) was used specifically for the acute MDP stimulation experiment as an orthogonal approach to enable unbiased enumeration of proliferating intestinal cells following ligand exposure. These clarifications have been added throughout the revised manuscript to ensure consistency and accessibility.

Figure 4IJ text "the absolute barrier size" does not appear to be related to the presented analysis. Additionally the area of analysis outlined in Fig 4I and the methods suggests

pneumatic duct-derived lumen mucins would be included in the quantification. I think the most pertinent measure would be goblet cell number/density rather than total mucins, which would be more variable from peristalsis and handling.
This is an important point by reviewer 1 and we agree that our original wording overstated what this assay measures. We have revised the Results text to remove the phrase “absolute barrier size” and now refer specifically to Alcian blue-positive mucin area. We also explicitly clarify in the Results and Methods that Alcian blue staining detects secreted mucins rather than goblet cells themselves, and therefore this assay reports mucin-positive area, not goblet cell number or density. Because whole-mount Alcian blue staining does not reliably resolve individual goblet cells, we do not believe goblet cell counting would be technically accurate with this dataset. We have adjusted the interpretation accordingly and now frame these measurements as changes in mucin output/coverage rather than goblet cell abundance. These revisions improve accuracy and prevent over-interpretation of the staining readout.

Figure 5: the qPCR data in 5HI suggest the vtg signal in panels A-G was driven by the 3 females to 2 males split in the scRNAseq dataset. Can the authors subanalyse the individual scRNAseq animals by male/female split to confirm this?
We appreciate this thoughtful suggestion. Unfortunately, this specific re-analysis is not technically feasible with the current dataset. For the scRNA-seq experiment, intestines from multiple fish were dissociated and pooled prior to library preparation, so individual cells cannot be traced back to a specific fish. As a result, we cannot stratify the existing single-cell dataset by animal or sex post hoc. In addition, there is currently no validated, intestine-specific gene expression signature that reliably distinguishes male and female zebrafish at the single-cell level, which further limits computational sex assignment from gut scRNA-seq data alone.

Conclusion/Discussion text
The second clause in the final results paragraph is a bit overstated, the authors did not explore the effect of E2 exposure on cell death phenotype, admittedly this was only explored in adult mutants so there is the *in zebrafish larvae caveat here.
We agree that the original phrasing was too broad. Our E2 exposure experiments were performed in zebrafish larvae and did not directly test the adult epithelial cell death phenotype. We have therefore revised the sentence to narrow the claim and specify scope. The text now states that E2 exposure recapitulates several key NOD2-deficiency phenotypes in zebrafish larvae, rather than all phenotypes associated with NOD2 deficiency. This change better aligns the wording with the data presented and avoids overgeneralization.

Would the final clause "...key modifier of the disruptions..." be clearer as "estrogen and NOD2 oppose each other"?
We thank the reviewer for this helpful suggestion and agree that this wording improves clarity. We have revised the sentence accordingly to state that estrogen signaling and NOD2 act in opposing directions in the regulation of intestinal homeostasis. This change makes the mechanistic interpretation more explicit and avoids overly broad modifier language.

Discussion paragraph beginning "Further supporting a mechanistic link": this would be an appropriate place to discuss the zebrafish paper using tamoxifen vs M. marinum infection (PMID 36475748).
We are grateful to the reviewer for pointing us to this relevant study. We have revised the Discussion paragraph beginning “Further supporting a mechanistic link” to incorporate the zebrafish tamoxifen-*Mycobacterium marinum* infection paper (PMID 36475748). We now cite this work alongside the bazedoxifene study and use it to broaden the context for how pharmacologic estrogen receptor modulators influence host–microbe and inflammatory responses in zebrafish. We also updated the surrounding text to better reflect the expanding literature on estrogen signaling and gut immune

regulation across model systems. This addition strengthens the cross-model support for an estrogen-immune-epithelial interaction framework relevant to our NOD2 findings.

Rev. 2:
This is an interesting manuscript that demonstrates a new role for estradiol and NOD2 signaling in intestinal cell homeostasis. The integration of scRNAseq and phenotypic characterization provides a multifaceted view of intestinal homeostasis, and the exploration of estrogen receptor involvement (even without discovering which estrogen receptors are involved) adds valuable mechanistic insight. The claims are novel and the conclusions are generally supported by the data.
We are grateful to reviewer two for their interest in the mechanistic insights provided by this study.

However, there are several limitations and areas where it is not clear whether the conclusions are supported by the data. The manuscript does not consistently control for sex across experiments, which may confound interpretation of sex-dependent effects. Key findings, such as those related to cell proliferation in adult intestine, would benefit from stratification or explicit reporting by sex.
Here, reviewer two raises an important point that we did not address adequately in our initial manuscript. In the revised version, we performed sex-stratified analysis of adult intestinal proliferation and added these results to the revised manuscript. Specifically, WT and *nod2*$^{-/-}$ proliferation measurements are now reported separately for males and females, and the stratified counts are included in the updated figure panel and legend. The sex-stratified analysis supports the overall genotype-dependent proliferation differences while also clarifying sex-associated effects. We appreciate the reviewer's recommendation, which has improved the rigor and clarity of the study.

Some data presentations, including quantification of immune and proliferative cell populations, would be more rigorous if shown as super plots to illustrate animal-level and tissue-/cell-level variability.
We appreciate the reviewer's interest in conveying animal-level and tissue-level variability. In our analyses, the biological replicate is the individual fish, and all statistical tests were performed using per-animal summary values derived from multiple images or regions of interest per intestine. Thus, animal-level variability is already represented in our plots, where each point corresponds to an individual animal. Tissue-level sampling variability was controlled by standardized ROI definitions, consistent acquisition settings, blinded quantification, and averaging of multiple fields per intestine prior to statistical testing.
Although rare, we concur that superplots have a visual appeal. However, they do not change the underlying statistical unit or test outcome. Because our conclusions are based on per-animal statistics rather than per-image or per-cell statistics, converting these datasets to superplots would not have any effect on the results or their interpretation.
In response to related reviewer suggestions regarding spatial rigor, we have strengthened the analysis of immune cell distributions by adding compartment-resolved quantification of Lcp1^{+} leukocytes along the intestinal fold axis (tip, mid, basal, stromal/lamina propria). This new apical-basal distribution analysis has been added to the manuscript (Fig. 2L and Results text) and provides additional biological resolution beyond total cell counts alone. This directly addresses variability in tissue-level localization and supports the reported genotype-dependent differences in immune cell accumulation.
Together, we believe the current data presentation, combined with clarified replicate definitions and the new spatial Lcp1 analysis, provides a rigorous and transparent representation of both animal-level and tissue-level effects.

Finally, further clarity on mechanism of estrogen signaling could be achieved by including additional pharmacologic controls and exploring the roles of both nuclear and membrane estrogen receptors using specific agonists/antagonists.

We thank the reviewer for this constructive suggestion and agree that receptor-selective pharmacologic and genetic dissection would provide additional mechanistic resolution. However, we believe these experiments are beyond the scope of the present study. Our objective here was to establish and functionally validate the presence of a NOD2-estrogen interaction in intestinal homeostasis using complementary transcriptomic and *in vivo* functional assays. Across multiple independent readouts, estradiol and tamoxifen produced consistent and directionally concordant effects on epithelial proliferation, goblet cell-associated mucin output, immune cell recruitment, and injury sensitivity, supporting a robust estrogen-dependent modifier effect.
As this study represents a comparatively novel set of results, we have attempted to be very precise in acknowledging unanswered mechanistic questions in the Limitations section, where we state that estradiol and tamoxifen may engage additional pathways, that the responsible estrogen receptor isoform remains unconfirmed, and that receptor-specific approaches, including CRISPR editing of estrogen receptor paralogs and selective receptor modulators, will be required to resolve pathway-level mechanism. We have framed these experiments as important future directions. We hope the reviewer agrees that this clarification appropriately defines the mechanistic boundaries of the current work while identifying the most informative next steps.

Note that I do not have the expertise to analyze the scRNAseq data in detail. GSE299263 is deposited on NCBI (scheduled for public release in March 2026) but I was not given a reviewer token to access it.
We apologize for this oversight. The reviewer access token was inadvertently omitted at submission. We have now generated and provided the reviewer access credentials for GSE299263 so the dataset can be accessed during peer review. We appreciate the opportunity to correct this and will ensure reviewer tokens are included with future submissions.

Specific comments:
1. Is sex being controlled for? In some cases it is not mentioned (eg Fig 1B), in other cases it is purposely confounded (eg scRNAseq experiments on 2M, 3F, with results pooled/not controlled for sex, Fig 2). Are there any sex-dependent effects of either NOD2 mutant phenotypes or estrogen exposure phenotypes in the gut? If the authors are focused on NOD2 effects in common to both sexes, or that occur in either sex (without regards to potential sex differences), then please make this clear for each and every experiment.
We thank the reviewer for highlighting the importance of sex as a biological variable in our study. We have revised the manuscript throughout to explicitly document sex and developmental stage for each experiment in the Methods, Results, and Legends.
In this study, the manuscript organization reflects the chronological trajectory of discovery. Early baseline phenotyping and transcriptomic experiments were performed using mixed-sex in-crossed sibling cohorts to define core genotype-dependent intestinal phenotypes shared across animals, consistent with standard practice for initial genotype characterization. Larval experiments were conducted prior to sexual differentiation and are therefore not confounded by sex.
Following single-cell transcriptomic profiling, we observed a strong estrogen-responsive signature in *nod2*-mutants, which identified sex and hormonal context as important biological modifiers. We therefore performed targeted follow-up experiments with sex recorded and analyzed explicitly. These now include sex-stratified adult epithelial proliferation (PCNA) analysis (Fig S6) and sex-stratified epithelial and immune phenotypes (Anxa4, Lcp1, TUNEL) (Fig S7). This is in addition to the sex-separated intestinal vtg qPCR validation (Fig 5H-I)
To further improve clarity for readers, we have also added developmental stage icons (larval vs adult) directly to each figure panel to visually indicate the life stage analyzed. This allows rapid identification of whether data derive from larvae (pre-sex differentiation) or adult animals.
We also added text in the Results and Discussion clarifying when experiments were mixed-sex vs sex-stratified, and expanded the Limitations section to note that not all early experiments were originally powered for sex-specific analysis. Future work will use prospectively sex-balanced designs.

2. Related, NOD2-deficiency increased the estrogen response (vtg gene expression) in adult females but not in males (Fig 5). Does this suggest that NOD2 functions to regulate intestinal

homeostasis in females but not in males? How is this result reconciled with previous results from the manuscript (eg PCNA-pos cells) in wt vs nod2 mutant adults? Are the PCNA results in Fig 3 containing only females? Or mixed sex but the female signal swamps the male signal? It would be interesting (and add to the rigor and comprehensiveness of the manuscript) if the authors could present data in Fig 1-4 with regard to sex. For example, in Figure 4G, does the goblet cell number variability and/or association with genotype change when graphed according to sex?

This is an important point that we address with new analyses and clarifying text in our revised manuscript. Our revised analyses indicate that NOD2 regulates intestinal homeostasis in both sexes, but that the magnitude of several phenotypes is greater in females, particularly for estrogen-responsive transcriptional outputs. Specifically: vtg gene induction is female-biased (Fig 5H-I), adult epithelial proliferation defects (PCNA) occur in both sexes, with stronger effect size in females (Fig S6), and secretory lineage loss, leukocyte accumulation, and apoptosis are observed in both sexes, again with greater magnitude in females (Fig S7)

Earlier baseline adult phenotyping panels (Figs 1-4) were performed using mixed-sex cohorts to define shared genotype-dependent phenotypes. We now state this explicitly in the Results, Methods, and Legends. The new sex-stratified analyses demonstrate that these genotype effects are not driven solely by sex but rather are present across sexes with sex-modified effect sizes.

We have revised the Discussion to clarify that we interpret NOD2 as a regulator of shared epithelial and immune programs whose downstream phenotypic magnitude is modified by hormonal context, rather than as a female-specific regulator. We also note that the NOD2-estrogen interaction axis represents a broader regulatory framework that is the subject of ongoing dedicated work beyond the scope of the present manuscript.

3. NOD2-deficient intestines contain larger population of dysregulated immune cells vs wt. Fig 2K - nod2 mutants have greater number of Lcp1+ cells. This conclusion is not supported by the data (as presented) - could the authors show these data as a super plot or similar (PMID 32346721), where we could see the number of Lcp1+ cells per fish (unit of biological replicate) and the number of Lcp1+ cells per um or ROI (tissue-level variability)?

We respectfully disagree with the assertion that these conclusions are not supported by the data as presented or that superplot visualization is required for validity. Superplots are one useful visualization approach for hierarchical data, but they are not a methodological requirement and are not the only acceptable way to display replicate structure. The strength of the conclusions derives from the experimental design, replicate definition, and statistical testing.

In all figures, the biological unit of replication, sampling framework, and statistical tests are explicitly defined and appropriate to the experimental design. Statistical analyses were performed at the correct biological replicate level, and the reported significance and effect sizes are based on those replicate-level values. Changing the visualization to a superplot would not alter the underlying data, statistical outcomes, or conclusions.

Importantly, several of the figures already display biological replicates directly (for example, one point per intestine in EdU and Alcian blue analyses), and others use ROI/villus-level measurements that are standard and widely accepted for histological quantification when coupled with replicate-level statistics, which is how our data were analyzed. We have further strengthened the manuscript by clarifying replicate definitions, ROI sampling strategy, and statistical units in the revised legends and Methods to remove any ambiguity. Our conclusions are supported by the data and the replicate-level statistical analysis as presented.

4. An interesting result from the scRNAseq is the upregulation of estrogen-responsive genes in nod2 mutants vs wt. The vtg genes suggest the involvement of nuclear estrogen receptors (esr1, esr2a, esr2b), but shouldn't the manuscript also consider the possibility that a membrane estrogen receptor, eg gper1, might also be involved? For example, in Fig 5, can the manuscript show the expression of gper1 in intestine, and how it might vary by cell type in wt vs nod2 mutants?

We thank the reviewer for this suggestion. We agree that membrane estrogen signaling via Gper1 is an important pathway to consider alongside nuclear estrogen receptors. We therefore re-examined

our intestinal scRNA-seq dataset specifically for *gper1* expression across epithelial, immune, and stromal compartments in both WT and nod2 mutant intestines. In our dataset, *gper1* transcripts were not detected in any intestinal cell population in either genotype, preventing meaningful assessment of its contribution in this context. This is consistent with prior reports that *gper1* expression in zebrafish gut tissue is very low relative to nuclear estrogen receptors.

To address this point directly, we have added clarifying text to the Discussion and Limitations sections noting that membrane estrogen receptor signaling remains a plausible additional pathway, but could not be evaluated here due to lack of detectable intestinal *gper1* expression in our single-cell dataset. We further state that resolving receptor-specific mechanisms will require targeted genetic or pharmacologic approaches in future work.

5. Related, tamoxifen is a selective estrogen receptor modulator, meaning it acts as an agonist in some cells but an antagonist in others. Are the authors arguing here that tamoxifen is acting as an estrogen receptor antagonist? In that case, Fig 6 is missing a key control - what happens in wt larvae exposed to vehicle (shown) vs E2 (shown) vs tam (shown) vs E2+tam (not shown)? Because the authors are not taking a genetic approach (eg exposing esr or gper mutants), it would add additional rigor to the manuscript if these experiments could be repeated with an additional estrogen receptor antagonist, such as ICI182,780 (an anti-estrogen, not a selective estrogen receptor modulator). If the results are consistent, this would more clearly demonstrate a role for estrogens and for nuclear estrogen receptors. Alternatively, if the results are discordant, this could suggest a role for GPER, which could be further tested using GPER-specific agonists/antagonists that have low/no affinity for nuclear estrogen receptors.

We appreciate this thoughtful and mechanistically insightful comment. In this study, we interpret tamoxifen primarily as functionally antagonizing estrogen signaling in the larval intestine under our experimental conditions, based on the directionality of phenotypes, where tamoxifen opposes estradiol-driven effects across multiple readouts (immune cell recruitment, epithelial proliferation, and mucin output), and our transcriptional evidence that estrogen-responsive programs are engaged in *nod2*-deficient guts. We have also expanded the discussion and results section to include PMID 36475748 (brought to our attention by Reviewer one), a manuscript that provides genome-wide transcriptional evidence that tamoxifen acts as an estrogen receptor antagonist in larval zebrafish. That said, we acknowledge that tamoxifen cannot be assumed to behave as a “pure” antagonist in all cell types.

Regarding the suggested controls and additional pharmacology: we agree that estradiol plus tamoxifen, alongside the use of an additional anti-estrogen such as ICI182,780 would further refine receptor-level mechanisms. However, we consider these additions beyond the scope of the current manuscript, which is focused on establishing the existence and physiological relevance of a NOD2-estrogen interaction in intestinal homeostasis using multiple complementary functional assays. We also note practical constraints in expanding pharmacologic arms in this system, as tamoxifen combined with DSS caused lethality in WT larvae, limiting the feasibility of extended combinatorial dosing under injury conditions (as noted in our Limitations section).

To address the reviewer’s core concern, we have clarified in the text that tamoxifen is a SERM and that its effects here are interpreted (and supported by PMID 36475748) as functional antagonism in the intestine, while explicitly noting that receptor specificity remains unresolved. We further outline receptor-selective antagonists/agonists (including ICI182,780) and/or genetic approaches (*esr* perturbation) as important future directions to distinguish nuclear versus membrane estrogen receptor contributions.

6. Limitations section - the NOD2 mutant phenotypes are compelling, but it would be more rigorous to mention, as a limitation, that the phenotypes you see could be hypomorphic and not due to a true null allele (eg, even in the absence of NOD2 protein, there could be transcriptional adaptation occurring, see for example PMID 39939773, 31951195, 40128410, 30944477, where the nod2 mutant RNA is degraded and triggers upregulation of compensatory genes that can partially compensate for loss of NOD2 protein). This is supported by the MDP experiments (NOD2 ligand) where there is still a small effect in nod2 mutants. Doing additional experiments to test for the presence of transcriptional adaption

will NOT change the manuscript's conclusions and is not necessary; adding a sentence or two in the limitations acknowledging the possibility of compensation is sufficient.
We thank the reviewer for this thoughtful suggestion and agree that this is an important caveat. We have revised the Limitations section to explicitly acknowledge that our CRISPR frameshift allele, while predicted to severely disrupt NOD2 function, could behave as a hypomorphic allele *in vivo* due to transcriptional adaptation or compensatory gene upregulation triggered by mutant transcript degradation. We now note that such compensation has been described for other CRISPR-induced alleles and that the small residual response to MDP observed in *nod2* mutants is consistent with possible partial pathway activity or compensation. We further clarify that although this possibility does not change the interpretation of our central conclusions, it represents an important consideration for allele-level mechanistic interpretation. This text has been added to the Limitations section as recommended.

7. Fig 1B - shorter gut length phenotype in NOD2 mutants. Is gut length a function of fish length (eg standard length, as in PMID 19891001)? Could the reduced gut length in the 9 NOD2 mutants examined really be due to those fish being shorter (eg not controlling for standard length) and not a function of genotype?
Here, reviewer two raises an interesting point that we had not previously considered. In the revised manuscript we addressed this question by incorporating standard body length measurements and size-normalized analyses. Specifically, we measured standard body length (snout to base of caudal fin) for all fish used in the gut-length analysis. We find that WT and *nod2*$^{-/-}$ adults do not differ significantly in standard body length or body weight. In contrast, total intestinal length remains significantly reduced in *nod2*$^{-/-}$ fish. To further control for body size, we added normalized metrics in which gut length is divided by standard body length and body weight. In both cases, the reduction in gut length in *nod2*$^{-/-}$ mutants remains significant after normalization. These new data are now included in Fig 1 (body length and normalized gut-length panels), described in the Results, and detailed in the Methods section (fish weight, body length, and gut length measurements). Together, these analyses demonstrate that the reduced gut length phenotype is not explained by differences in overall fish size and instead reflects a genotype-associated intestinal phenotype. We feel these observations are interesting in the context of interorgan communications and thank reviewer two for the suggestion.

8. Fig 3G, 3H: In panel H, EdU-pos cells were quantified using flow cytometry, why were they not quantified this way in panel G? How were EdU-pos cells quantified in panel G?
We agree that our original manuscript did not clearly explain the rationale and implementation of the different quantification approaches. We have revised both the Results text and the Fig. 3 legend to explicitly state how EdU^{+} cells were quantified in each panel and why different methods were used. For Fig. 3G (conventionally reared vs germ-free comparisons), EdU^{+} cells were quantified from whole-mount intestines by confocal imaging and counting EdU^{+} nuclei within a standardized intestinal region of interest (ROI), which preserves tissue architecture and enables consistent ROI-based quantification across rearing conditions. For Fig. 3H (acute MDP stimulation in germ-free larvae), we used flow cytometry of dissociated intestines as an orthogonal, quantitative approach to enable unbiased enumeration of cycling intestinal cells following ligand exposure; due to throughput and the pooled-sample design (15 intestines per data point), this assay is well suited to the acute stimulation experiment. These clarifications have been added to the Results section and explicitly incorporated into the Fig. 3G/H legend text to prevent confusion.

9. Fig 3J: the conclusion that nod2 mutants have reduced cell proliferation in intestine (PCNA-pos cells) is not supported by the data (as presented). Could the authors show these data as a super plot or similar (PMID 32346721), where we could see the number/percent of PCNA+ cells per fish (unit of biological replicate) and the number/percent of PCNA+ cells per um or ROI (tissue-level variability)?
10. Same comment for Suppl Fig 5 (see #9)
11. Analogous comment for graphs in Fig 4 (see #9)

Points 9-11 reiterate point 3, requesting that we change graphs from the commonly used box plots to the rarely seen superplots based on PMID 32346721, a non-peer-reviewed viewpoint published in 2020. As stated above, we concur that such plots have an amount of visual appeal. However, we also feel it important to reiterate that the use of superplots is exceedingly rare, including apparently by the authors of PMID 32346721, (e.g. PMID 41279105, PMID 41549998, PMID 40690378). Additionally, PMID 32346721 rightfully demonstrates that superplots are purely visual tools that do not affect the outcomes of statistical evaluation of data. We have gone to considerable lengths throughout this study to ensure that we applied the necessary degrees of rigour in our generation and evaluation of all data in this manuscript and, working with all reviewers, we made extensive revisions to all sections of our manuscript to clarify how each experiment was performed. For those reasons, we do not feel that changing all graphs of this study to superplots will enhance the quality or significance of our work.

12. Methods - please provide more details on how fish were housed singly (Fig 1I-M). Do fish in different tanks housed on the same system sharing recirculating water have different bacterial community composition in their guts?
We thank the reviewer for highlighting the need for clearer housing detail. We have revised the Methods to more explicitly describe the single-housing condition. WT and *nod2*$^{-/-}$ adults in the "single-housed" group were maintained in physically separate tanks on the same recirculating water system for ≥8 weeks prior to sampling, under matched water quality parameters, feeding schedule, stocking density, and husbandry practices. We now explicitly state that although system water was shared, tank-level separation results in distinct local microbial environments (including tank biofilms and host-driven microbial shedding), which can permit divergence in gut microbial communities. We also clarified this point in the Results text to avoid implying system-level isolation. These additions improve transparency of the housing design and interpretation of the microbiome comparisons.

Rev. 3:
The manuscript by Eklund and Foley represents an impressive body of work that advances understanding of the function of the IBD-associated NOD2 gene function in the vertebrate intestinal epithelium. Using single cell RNAseq, the authors identify a role for NOD2 in repression of estrogen-response genes. They go on to test the functional relevance of this mis-regulation using pharmacological approaches that support a role for excessive estrogen-response gene expression in NOD2-associated intestinal pathology. Specific comments on the manuscript are listed below.

1. A key assay throughout the paper is the quantification of cell proliferation in the intestinal epithelium. Confusingly, the authors use two different assays and three different representations of their data. In some instances they quantify EdU+ cells by immunohistochemistry, and either report this as the number of EdU+ cells/gut (preferable, because the values are easy to understand) or as a percentage in Fig 3G (presumably percentage of all epithelial cells?). They should use a consistent scoring method throughout. They should also provide more explanation of how this quantification is actually performed. Do they reconstruct the volume of the gut tube? What region of the gut is quantified (the representative images for Fig 3F looks different from that of Supplemental Fig 5 or Fig 6A and C)? For Fig 3H they report a different assay for quantifying EdU+ cells by flow cytometry. What is the reason for using this different assay in this one instance?
We thank the reviewer for highlighting this point and agree that the original text did not sufficiently explain the rationale and implementation of the different proliferation assays. We have revised the Results, Methods, and figure legends to clarify both the methodology and the reporting metrics. Imaging-based EdU quantification was performed using whole-mount confocal microscopy, with EdU$^+$ nuclei counted either across the entire intestine (baseline genotype comparisons; Supplementary Fig. 5) or within a standardized intestinal region of interest (ROI) (rearing-condition comparisons; Fig. 3G). In ROI-based analyses, EdU$^+$ cells are reported as a percentage of total epithelial nuclei in the quantified region. We now explicitly state the quantified region, denominator,

and counting approach in both the Methods and figure legends. Flow cytometry (Fig. 3H) was used specifically for the acute MDP stimulation experiment as an orthogonal approach to enable unbiased enumeration of proliferating intestinal cells following ligand exposure. These clarifications have been added throughout the revised manuscript to ensure consistency and accessibility.

2. Another key assay is the quantification of goblet cells. For this they report quantifying the Alcian positive area in a region of interest. This would involve setting some threshold of blue intensity to discriminate background from signal. How was this done in an unbiased manner?
We agree that the thresholding and segmentation procedure required clearer description. We have revised both the Results and Methods sections to explicitly detail how Alcian blue quantification was performed in an unbiased and reproducible manner. Briefly, all samples were imaged using identical illumination and exposure settings. Image analysis was performed blinded to genotype and treatment. A single fixed color threshold for Alcian blue signal was established using control images and then applied uniformly across all samples within each experiment without per-image adjustment. The intestinal region of interest (ROI) was defined using consistent anatomical landmarks (hepatic bend to cloaca), excluding extra-intestinal tissue. Alcian blue-positive area and total ROI area were then quantified using Fiji (ImageJ), and values are reported as percent Alcian blue-positive intestinal area. These clarifications have been added to the Methods and figure-associated Results text to improve transparency and reproducibility.

3. The results switch between adult and larval experiments extensively. It would be helpful for the authors to explain the motivation for performing experiments at these specific stages. It would be helpful in the figures to include larval or adult icons.
We agree with reviewer three that it's important point to clarify the decision-making processes that led us to work with larvae or adults. We have now clarified both the rationale for using larval versus adult zebrafish and the developmental stage associated with each experiment throughout the revised manuscript. We added explanatory text in the Methods sections describing why different developmental stages were used for different assay types. In brief, larval models were used where optical transparency, whole-mount imaging, germ-free derivation, and short-term pharmacologic manipulation are required, whereas adult models were used to assess mature intestinal architecture, long-term epithelial homeostasis, microbiome composition, and sex-dependent phenotypes. To further improve clarity and figure-level interpretability, we added developmental stage icons (larval vs adult) to all main and supplementary figures. Corresponding figure captions now explicitly state that the icon indicates the developmental stage at which the experiment was performed. Together, these changes make the experimental staging transparent and provide readers with both textual and visual cues to distinguish larval and adult datasets across the study.

4. The authors cite two other independently generated nod2 deficient zebrafish. They should provide more explanation of how the different mutants were made and they similarities and differences in the phenotypes reported.
We are grateful to reviewer three for this helpful suggestion. We have expanded the early Discussion to more clearly compare prior zebrafish *nod2* loss-of-function models with our allele. We now distinguish the initial larval study using splice-blocking morpholinos targeting the *nod2* exon 1 donor site, which demonstrated conserved Nod pathway expression and impaired antibacterial defense (including reduced *duox* expression and decreased control of systemic bacterial infection), from a later study that generated CRISPR/Cas9 *nod2* mutant lines targeting distinct coding regions and reported intestinal phenotypes such as gut shortening, leukocyte accumulation, DSS sensitivity, and inflammatory remodeling. We clarify that our line is an independently generated CRISPR-induced frameshift allele predicted to severely disrupt NOD2 function.

5. Humans do not typically harbor complete deletions of NOD2, as in this zebrafish model. The authors should discuss how their results with nod2 deficient zebrafish fit into what is known about the spectrum of NOD2 alleles in humans that are associated with disease.

We agree with the reviewer and have revised the opening section of the Discussion to better position our zebrafish *nod2* null model within the spectrum of human and rodent NOD2 alleles. While complete gene deletion is uncommon in humans, the most prevalent Crohn's disease-associated NOD2 variants are coding alleles, including frameshift and missense mutations, that are widely considered functional loss-of-signaling or strongly hypomorphic with respect to MDP sensing and downstream NF-κB activation. We now explicitly note that rodent models likewise include both null and partial-loss Nod2 alleles that produce overlapping barrier and inflammatory phenotypes. The revised text clarifies that our CRISPR frameshift zebrafish allele represents the severe end of the functional spectrum and is intended to define core NOD2-dependent homeostatic mechanisms, whereas human disease more often reflects partial impairment combined with additional genetic and environmental modifiers. This contextual framing has been added to the first paragraphs of the Discussion to improve translational interpretation.
