# Supplementary material for: Estrogen impacts NOD2-dependent regulation of intestinal homeostasis
Source: PLoS Biol. 2026 Jun 9;24(6):e3003766. doi: 10.1371/journal.pbio.3003766 (PMC13271492; doi:10.1371/journal.pbio.3003766)
Supplement: Attachment — Submitted filename: 26_Response_To_Reviewers_02.pdf [file pbio.3003766.s027.pdf]

UNIVERSITY OF BATH
Bath BA2 7AY • United Kingdom
Prof Edan A. Foley
GSK Chair in Biomedical Microbiomics
Email ef796@bath.ac.uk

Dear Editors of PLoS Biology,

We would like to take this opportunity to thank you and the reviewers for a supportive and constructive assessment of our manuscript, Estrogen Impacts NOD2-Dependent Regulation of Intestinal Homeostasis.

In our revised manuscript, we have addressed the remaining points raised by Reviewer 2. In particular, we have explicitly defined the unit of biological replication across experiments and revised the Methods and figure legends to ensure transparency in how data were quantified and analyzed. We have also expanded the Discussion to more clearly articulate the context-dependent activity of tamoxifen as a selective estrogen receptor modulator, including its potential agonist and antagonist effects and how this may influence interpretation of our findings.

We are grateful to the reviewer for raising these points, and concur that the changes to the text have added important clarity and context.

Thank you again for your time and consideration. We would be happy to provide any additional information if needed.

Sincerely,

Edan Foley

Edan A. Foley

Reviewers' comments

Rev. 2: I commend the authors for their response to reviewer comments. I think the revised manuscript is clearer and more rigorous. Two points which I think could be addressed further:

We would like to thank reviewer two for their support, but also for their feedback throughout the review process. Their comments clearly added to the manuscript, and we appreciate their interest in our research.

(1) I'm still unsure about the unit of biological replication in some experiments, which is why I originally suggested using SuperPlots. For instance, Figure 2K aims to show that nod2 mutants have more Lcp1+ cells than wild-type fish. The legend states that each group consists of five fish, yet the graph displays dozens of data points. If a single fish is considered one replicate, shouldn't there only be five data points per group? My earlier SuperPlot suggestion was intended to have the authors add these five fish-level data points to the existing graph, allowing readers to see both the Lcp1+ cells per intestine/ROI and per fish simultaneously. Currently, each dot represents a measurement from a fish intestine image. Is this what counts as a replicate? If so, what is the reasoning, and why not use Lcp1+ cell counts per fish instead? After reviewing the methods section (see below), I still couldn't find clarification. Could the authors explicitly state what constitutes a unit of replication? While variability in results is expected (and reflects underlying biology), choosing different definitions for the unit of replication could impact the conclusions regarding differences between wild type and nod2 mutants.

"Quantification of Anxa4^{+} secretory cells, Lcp1^{+} leukocytes, and TUNEL^{+} apoptotic cells was performed on sagittal intestinal sections using Fiji (ImageJ). Individual villi were manually outlined using the DNA (nuclear) channel to define the epithelial region of interest. Within each villus ROI, Anxa4^{+}, Lcp1^{+}, and TUNEL^{+} cells were detected using a fixed threshold applied uniformly across all samples and counted manually using the cell counter tool. Total epithelial cell number per villus was determined by counting all DNA^{+} nuclei within the same ROI. To account for differences in villus size and cellular density between sections and fish, data were expressed as the percentage of marker-positive cells per villus, calculated as (number of Anxa4^{+}, Lcp1^{+}, or TUNEL^{+} cells ÷ total number of DNA^{+} nuclei within the ROI) x 100."

Thank you for raising this important point regarding the unit of biological replication. We apologize for the lack of clarity in the original submission. In these experiments, the biological replicate is the individual fish (n=5 per group). Multiple villi (regions of interest, ROIs) were analyzed per fish to capture intra-intestinal variability. In the original figures, each dot represented a single villus measurement, which may have given the impression that these were independent biological replicates. We have now revised the Methods section to explicitly define the unit of replication and clarify that measurements from individual villi are averaged per fish for statistical analysis, with each fish treated as a single biological replicate. The figure captions have also been updated. Importantly, statistical analyses are now explicitly described as being performed on fish-level averages, thereby avoiding pseudoreplication. We appreciate the reviewer's suggestion, which has improved the clarity and rigor of our data presentation.

(2) Tamoxifen is a selective estrogen receptor modulator, and I believe the discussion section should more clearly address in the 'limitations' section that the authors are assuming TAM acts solely as an antagonist (though they may have strong reasons for making this assumption). However, TAM can also function as an ER agonist, and it may act as a GPER agonist as well (see PMID 15705806, 16239258). The revised discussion

currently states, "…tamoxifen may engage additional target pathways." This wording seems somewhat vague and could be expanded to clarify that there is substantial evidence showing TAM can behave as an agonist in some tissues or contexts and as an antagonist in others, even within the same organism simultaneously, and how this might impact the interpretation of the author's results. This complexity helps explain why TAM cannot be used indefinitely in humans for treating ER-positive breast cancers. That said, I am not insisting on this point and will defer to the editor's judgment regarding how extensively this limitation should be addressed.

Thank you for this thoughtful and important comment. We agree that tamoxifen is a selective estrogen receptor modulator with context-dependent activity and does not function exclusively as an estrogen receptor antagonist. Tamoxifen has been shown to exhibit both agonist and antagonist properties depending on tissue context, receptor subtype, and co-regulator availability, and may also signal through non-classical pathways. We have revised the Discussion to explicitly acknowledge this complexity and clarify that future studies are required to definitively assess the involvement of each estrogen receptor isoform in the maintenance of intestinal homeostasis. We now note that tamoxifen can act as an agonist in certain contexts and that its mixed pharmacological activity may influence the observed phenotypes. This has been incorporated into the limitations section of the Discussion.
