# Supplementary material for: Estrogen impacts NOD2-dependent regulation of intestinal homeostasis
Source: PLoS Biol. 2026 Jun 9;24(6):e3003766. doi: 10.1371/journal.pbio.3003766 (PMC13271492; doi:10.1371/journal.pbio.3003766)
Supplement: Peer review history

# Editor report: Decision Letter 0

**DOI:** 10.1371/journal.pbio.3003766.r001

**Authors:** Ines Alvarez-Garcia

17 Nov 2025

Dear Dr Foley,

Thank you for submitting your manuscript entitled "Estrogen impacts NOD2-dependent regulation of intestinal homeostasis" for consideration as a Research Article by PLOS Biology.

Your manuscript has now been evaluated by the PLOS Biology editorial staff as well as by an academic editor with relevant expertise and I am writing to let you know that we would like to send your submission out for external peer review.

However, before we can send your manuscript to reviewers, we need you to complete your submission by providing the metadata that is required for full assessment. To this end, please login to Editorial Manager where you will find the paper in the 'Submissions Needing Revisions' folder on your homepage. Please click 'Revise Submission' from the Action Links and complete all additional questions in the submission questionnaire.

Once your full submission is complete, your paper will undergo a series of checks in preparation for peer review. After your manuscript has passed the checks it will be sent out for review. To provide the metadata for your submission, please Login to Editorial Manager (https://www.editorialmanager.com/pbiology) within two working days, i.e. by Nov 19 2025 11:59PM.

If your manuscript has been previously peer-reviewed at another journal, PLOS Biology is willing to work with those reviews in order to avoid re-starting the process. Submission of the previous reviews is entirely optional and our ability to use them effectively will depend on the willingness of the previous journal to confirm the content of the reports and share the reviewer identities. Please note that we reserve the right to invite additional reviewers if we consider that additional/independent reviewers are needed, although we aim to avoid this as far as possible. In our experience, working with previous reviews does save time.

If you would like us to consider previous reviewer reports, please edit your cover letter to let us know and include the name of the journal where the work was previously considered and the manuscript ID it was given. In addition, please upload a response to the reviews as a 'Prior Peer Review' file type, which should include the reports in full and a point-by-point reply detailing how you have or plan to address the reviewers' concerns.

During the process of completing your manuscript submission, you will be invited to opt-in to posting your pre-review manuscript as a bioRxiv preprint. Visit http://journals.plos.org/plosbiology/s/preprints for full details. If you consent to posting your current manuscript as a preprint, please upload a single Preprint PDF.

Feel free to email us at plosbiology@plos.org if you have any queries relating to your submission.

Kind regards,

Ines

--

Ines Alvarez-Garcia, PhD

Senior Editor

PLOS Biology

ialvarez-garcia@plos.org

# Peer review: Decision Letter 1

**DOI:** 10.1371/journal.pbio.3003766.r002

**Authors:** Ines Alvarez-Garcia

20 Jan 2026

Dear Dr Foley,

Thank you for your patience while your manuscript entitled "Estrogen impacts NOD2-dependent regulation of intestinal homeostasis" was peer-reviewed at PLOS Biology. Please also accept again my apologies for the delay in sending you our decision. The manuscript has now been evaluated by the PLOS Biology editors, an Academic Editor with relevant expertise, and by three independent reviewers.

The reviews are attached below. As you will see, the reviewers find the conclusions interesting and worth pursuing for publication, but they also raise several issues that will need to be addressed. Reviewer 1 asks for deeper histological analyses to show the regional effects, misses quantification in some of the experiments, and requests an analysis of the individual scRNAseq animals by male/female to confirm the 3 females to 2 males split in the dataset. Similarly, Reviewer 2 would like to know if sex is controlled for and thinks that some of the data should be presented according to sex. This reviewer also asks for a better presentation of the data to see animal-level and tissue-/cell-level variability and more mechanistic details on how estrogen signalling is achieved, among other issues. Reviewer 3 also requests clarifications on the methods and thinks that some of the results should be better discussed.

In light of the reviews, we would like to invite you to revise the work to thoroughly address the reviewers' reports. Given the extent of revision needed, we cannot make a decision about publication until we have seen the revised manuscript and your response to the reviewers' comments. Your revised manuscript is likely to be sent for further evaluation by all or a subset of the reviewers.

In addition to these revisions, you will need to complete some formatting changes, which you will receive in a follow up email. A member of our team will be in touch with a set of requests shortly.

We expect to receive your revised manuscript within 3 months. Please email us (plosbiology@plos.org) if you have any questions or concerns, or would like to request an extension.

At this stage, your manuscript remains formally under active consideration at our journal; please notify us by email if you do not intend to submit a revision so that we may withdraw it.

**IMPORTANT - SUBMITTING YOUR REVISION**

Your revisions should address the specific points made by each reviewer. Please submit the following files along with your revised manuscript:

1. A 'Response to Reviewers' file - this should detail your responses to the editorial requests, present a point-by-point response to all of the reviewers' comments, and indicate the changes made to the manuscript.

*NOTE: In your point-by-point response to the reviewers, please provide the full context of each review. Do not selectively quote paragraphs or sentences to reply to. The entire set of reviewer comments should be present in full and each specific point should be responded to individually, point by point.

You should also cite any additional relevant literature that has been published since the original submission and mention any additional citations in your response.

2. In addition to a clean copy of the manuscript, please also upload a 'track-changes' version of your manuscript that specifies the edits made. This should be uploaded as a "Revised Article with Changes Highlighted" file type.

3. Resubmission Checklist

When you are ready to resubmit your revised manuscript, please refer to this resubmission checklist: https://plos.io/Biology_Checklist

To submit a revised version of your manuscript, please go to https://www.editorialmanager.com/pbiology/ and log in as an Author. Click the link labelled 'Submissions Needing Revision' where you will find your submission record.

Please make sure to read the following important policies and guidelines while preparing your revision and fulfil the editorial requests:

a) *PLOS Data Policy*

Please note that as a condition of publication PLOS' data policy (http://journals.plos.org/plosbiology/s/data-availability) requires that you make available all data used to draw the conclusions arrived at in your manuscript. If you have not already done so, you must include any data used in your manuscript either in appropriate repositories, within the body of the manuscript, or as supporting information (N.B. this includes any numerical values that were used to generate graphs, histograms etc.). Please also indicate in each figure legend where the data can be found. For an example see here: http://www.plosbiology.org/article/info%3Adoi%2F10.1371%2Fjournal.pbio.1001908#s5

b) *Published Peer Review*

Please note while forming your response, if your article is accepted, you may have the opportunity to make the peer review history publicly available. The record will include editor decision letters (with reviews) and your responses to reviewer comments. If eligible, we will contact you to opt in or out. Please see here for more details:

https://blogs.plos.org/plos/2019/05/plos-journals-now-open-for-published-peer-review/

c) *Blot and Gel Data Policy*

Please provide the original, uncropped and minimally adjusted images supporting all blot and gel results reported in an article's figures or Supporting Information files. We will require these files before a manuscript can be accepted so please prepare them now, if you have not already uploaded them. Please carefully read our guidelines for how to prepare and upload this data: https://journals.plos.org/plosbiology/s/figures#loc-blot-and-gel-reporting-requirements

d) *Protocols deposition*

To enhance the reproducibility of your results, we recommend that if applicable you deposit your laboratory protocols in protocols.io, where a protocol can be assigned its own identifier (DOI) such that it can be cited independently in the future. For instructions see: https://journals.plos.org/plosbiology/s/submission-guidelines#loc-materials-and-methods

Thank you again for your submission to our journal. We hope that our editorial process has been constructive thus far, and we welcome your feedback at any time. Please don't hesitate to contact us if you have any questions or comments.

Sincerely,

Ines

--

Ines Alvarez-Garcia, PhD

Senior Editor

PLOS Biology

ialvarez-garcia@plos.org

------------------------------------

Reviewers' comments

Rev. 1: Stefan Oehlers – please note that this reviewer has signed the review.

Eklund & Foley use a novel zebrafish nod2 mutant allele to investigate the role of Nod2 in intestinal homeostasis, response to injury, and interaction with estrogen signalling. The manuscript's most novel finding is the interaction with estrogen signalling whereby Nod2 and E2/tamoxifen-regulated signalling appear to oppose each other. The discussion and limitation sections are quite clear about additional mechanistic experiments that *could* be performed to strengthen the manuscript's argument (such as use of genetics to target ER, additional small molecule probes). I am happy that sufficient experimental evidence is provided, most importantly in the form of a genetic tool combined with opposing small molecule probes with well characterised biological activity) to support the claims made in the manuscript and my comments are limited to deeper analysis of existing datasets and clarification points.

Specific comments

Some of the histological analyses would benefit from deeper analyses. The regional effects, while not essential to the narrative of the Nod2 story, are useful to advance the comparative intestinal biology field.

Figure 1BC: there is an intestinal fold height difference in the later figures (Fig 3I, S1. Maybe not so strong in 2IJ) that should be described early in the story here.

Figure 2K could be quantified to comment on the apical vs basal/LP location of leukocytes. From the example images it appears the increase in leukocyte density is driven by leukocytes at the base of the intestinal folds. It might be difficult to prove, but this would correlate with stroma>IEC expression of chemoattractant/adhesion genes in NOD2-/-.

Fig S5 and 3F: please be more clear about the location (along the intestinal bulb-cloaca axis) of tissue shown as examples and enumerated. I think the right edge of the S5 images are the cloaca but the framing of the 3F images is not immediately obvious.

Figure 3H: Can the authors explain why they switched to a flow cytometry methodology here? The whole mount imaging approach would allow detection of spatial patterns to NOD2/MDP-dependent proliferation that are missed by the analysis of dissociated cells.

Figure 4IJ text "the absolute barrier size" does not appear to be related to the presented analysis. Additionally the area of analysis outlined in Fig 4I and the methods suggests pneumatic duct-derived lumen mucins would be included in the quantification. I think the most pertinent measure would be goblet cell number/density rather than total mucins, which would be more variable from peristalsis and handling.

Figure 5: the qPCR data in 5HI suggest the vtg signal in panels A-G was driven by the 3 females to 2 males split in the scRNAseq dataset. Can the authors subanalyse the individual scRNAseq animals by male/female split to confirm this?

Conclusion/Discussion text

The second clause in the final results paragraph is a bit overstated, the authors did not explore the effect of E2 exposure on cell death phenotype, admittedly this was only explored in adult mutants so there is the *in zebrafish larvae caveat here.

Would the final clause "...key modifier of the disruptions..." be clearer as "estrogen and NOD2 oppose each other"?

Discussion paragraph beginning "Further supporting a mechanistic link": this would be an appropriate place to discuss the zebrafish paper using tamoxifen vs M. marinum infection (PMID 36475748).

Rev. 2:

This is an interesting manuscript that demonstrates a new role for estradiol and NOD2 signaling in intestinal cell homeostasis. The integration of scRNAseq and phenotypic characterization provides a multifaceted view of intestinal homeostasis, and the exploration of estrogen receptor involvement (even without discovering which estrogen receptors are involved) adds valuable mechanistic insight. The claims are novel and the conclusions are generally supported by the data.

However, there are several limitations and areas where it is not clear whether the conclusions are supported by the data. The manuscript does not consistently control for sex across experiments, which may confound interpretation of sex-dependent effects. Key findings, such as those related to cell proliferation in adult intestine, would benefit from stratification or explicit reporting by sex. Some data presentations, including quantification of immune and proliferative cell populations, would be more rigorous if shown as super plots to illustrate animal-level and tissue-/cell-level variability. Finally, further clarity on mechanism of estrogen signaling could be achieved by including additional pharmacologic controls and exploring the roles of both nuclear and membrane estrogen receptors using specific agonists/antagonists.

Note that I do not have the expertise to analyze the scRNAseq data in detail. GSE299263 is deposited on NCBI (scheduled for public release in March 2026) but I was not given a reviewer token to access it.

Specific comments:

1. Is sex being controlled for? In some cases it is not mentioned (eg Fig 1B), in other cases it is purposely confounded (eg scRNAseq experiments on 2M, 3F, with results pooled/not controlled for sex, Fig 2). Are there any sex-dependent effects of either NOD2 mutant phenotypes or estrogen exposure phenotypes in the gut? If the authors are focused on NOD2 effects in common to both sexes, or that occur in either sex (without regards to potential sex differences), then please make this clear for each and every experiment.

2. Related, NOD2-deficiency increased the estrogen response (vtg gene expression) in adult females but not in males (Fig 5). Does this suggest that NOD2 functions to regulate intestinal homeostasis in females but not in males? How is this result reconciled with previous results from the manuscript (eg PCNA-pos cells) in wt vs nod2 mutant adults? Are the PCNA results in Fig 3 containing only females? Or mixed sex but the female signal swamps the male signal? It would be interesting (and add to the rigor and comprehensiveness of the manuscript) if the authors could present data in Fig 1-4 with regard to sex. For example, in Figure 4G, does the goblet cell number variability and/or association with genotype change when graphed according to sex?

3. NOD2-deficient intestines contain larger population of dysregulated immune cells vs wt. Fig 2K - nod2 mutants have greater number of Lcp1+ cells. This conclusion is not supported by the data (as presented) - could the authors show these data as a super plot or similar (PMID 32346721), where we could see the number of Lcp1+ cells per fish (unit of biological replicate) and the number of Lcp1+ cells per um or ROI (tissue-level variability)?

4. An interesting result from the scRNAseq is the upregulation of estrogen-responsive genes in nod2 mutants vs wt. The vtg genes suggest the involvement of nuclear estrogen receptors (esr1, esr2a, esr2b), but shouldn't the manuscript also consider the possibility that a membrane estrogen receptor, eg gper1, might also be involved? For example, in Fig 5, can the manuscript show the expression of gper1 in intestine, and how it might vary by cell type in wt vs nod2 mutants?

5. Related, tamoxifen is a selective estrogen receptor modulator, meaning it acts as an agonist in some cells but an antagonist in others. Are the authors arguing here that tamoxifen is acting as an estrogen receptor antagonist? In that case, Fig 6 is missing a key control - what happens in wt larvae exposed to vehicle (shown) vs E2 (shown) vs tam (shown) vs E2+tam (not shown)? Because the authors are not taking a genetic approach (eg exposing esr or gper mutants), it would add additional rigor to the manuscript if these experiments could be repeated with an additional estrogen receptor antagonist, such as ICI182,780 (an anti-estrogen, not a selective estrogen receptor modulator). If the results are consistent, this would more clearly demonstrate a role for estrogens and for nuclear estrogen receptors. Alternatively, if the results are discordant, this could suggest a role for GPER, which could be further tested using GPER-specific agonists/antagonists that have low/no affinity for nuclear estrogen receptors.

6. Limitations section - the NOD2 mutant phenotypes are compelling, but it would be more rigorous to mention, as a limitation, that the phenotypes you see could be hypomorphic and not due to a true null allele (eg, even in the absence of NOD2 protein, there could be transcriptional adaptation occurring, see for example PMID 39939773, 31951195, 40128410, 30944477, where the nod2 mutant RNA is degraded and triggers upregulation of compensatory genes that can partially compensate for loss of NOD2 protein). This is supported by the MDP experiments (NOD2 ligand) where there is still a small effect in nod2 mutants. Doing additional experiments to test for the presence of transcriptional adaption will NOT change the manuscript's conclusions and is not necessary; adding a sentence or two in the limitations acknowledging the possibility of compensation is sufficient.

7. Fig 1B - shorter gut length phenotype in NOD2 mutants. Is gut length a function of fish length (eg standard length, as in PMID 19891001)? Could the reduced gut length in the 9 NOD2 mutants examined really be due to those fish being shorter (eg not controlling for standard length) and not a function of genotype?

8. Fig 3G, 3H: In panel H, EdU-pos cells were quantified using flow cytometry, why were they not quantified this way in panel G? How were EdU-pos cells quantified in panel G?

9. Fig 3J: the conclusion that nod2 mutants have reduced cell proliferation in intestine (PCNA-pos cells) is not supported by the data (as presented). Could the authors show these data as a super plot or similar (PMID 32346721), where we could see the number/percent of PCNA+ cells per fish (unit of biological replicate) and the number/percent of PCNA+ cells per um or ROI (tissue-level variability)?

10. Same comment for Suppl Fig 5 (see #9)

11. Analogous comment for graphs in Fig 4 (see #9)

12. Methods - please provide more details on how fish were housed singly (Fig 1I-M). Do fish in different tanks housed on the same system sharing recirculating water have different bacterial community composition in their guts?

Rev. 3:

The manuscript by Eklund and Foley represents an impressive body of work that advances understanding of the function of the IBD-associated NOD2 gene function in the vertebrate intestinal epithelium. Using single cell RNAseq, the authors identify a role for NOD2 in repression of estrogen-response genes. They go on to test the functional relevance of this mis-regulation using pharmacological approaches that support a role for excessive estrogen-response gene expression in NOD2-associated intestinal pathology. Specific comments on the manuscript are listed below.

1. A key assay throughout the paper is the quantification of cell proliferation in the intestinal epithelium. Confusingly, the authors use two different assays and three different representations of their data. In some instances they quantify EdU+ cells by immunohistochemistry, and either report this as the number of EdU+ cells/gut (preferable, because the values are easy to understand) or as a percentage in Fig 3G (presumably percentage of all epithelial cells?). They should use a consistent scoring method throughout. They should also provide more explanation of how this quantification is actually performed. Do they reconstruct the volume of the gut tube? What region of the gut is quantified (the representative images for Fig 3F looks different from that of Supplemental Fig 5 or Fig 6A and C)? For Fig 3H they report a different assay for quantifying EdU+ cells by flow cytometry. What is the reason for using this different assay in this one instance?

2. Another key assay is the quantification of goblet cells. For this they report quantifying the Alcian positive area in a region of interest. This would involve setting some threshold of blue intensity to discriminate background from signal. How was this done in an unbiased manner?

3. The results switch between adult and larval experiments extensively. It would be helpful for the authors to explain the motivation for performing experiments at these specific stages. It would be helpful in the figures to include larval or adult icons.

4. The authors cite two other independently generated nod2 deficient zebrafish. They should provide more explanation of how the different mutants were made and they similarities and differences in the phenotypes reported.

5. Humans do not typically harbor complete deletions of NOD2, as in this zebrafish model. The authors should discuss how their results with nod2 deficient zebrafish fit into what is known about the spectrum of NOD2 alleles in humans that are associated with disease.

# Author comment: Author response to Decision Letter 2

**DOI:** 10.1371/journal.pbio.3003766.r003

17 Feb 2026

Attachment Submitted filename: 26_NOD2_Response_To_Reviewers.pdf

# Peer review: Decision Letter 2

**DOI:** 10.1371/journal.pbio.3003766.r004

**Authors:** Ines Alvarez-Garcia

20 Mar 2026

Dear Dr Foley,

Thank you for your patience while we considered your revised manuscript entitled "Estrogen impacts NOD2-dependent regulation of intestinal homeostasis" for publication as a Research Article at PLOS Biology. This revised version of your manuscript has been evaluated by the PLOS Biology editors, the Academic Editor and one of the original reviewers.

Based on the reviews (attached below), we are likely to accept this manuscript for publication, provided you satisfactorily address the remaining points raised by Reviewer 2. Regarding the tamoxifen discussion (Point 2), you could simply modify the sentence to say that TAM can sometimes act as an agonist. Please also make sure to address the data and other policy-related requests stated below my signature.

As you address these items, please take this last chance to review your reference list to ensure that it is complete and correct. If you have cited papers that have been retracted, please include the rationale for doing so in the manuscript text, or remove these references and replace them with relevant current references. Any changes to the reference list should be mentioned in the cover letter that accompanies your revised manuscript.

In addition to these revisions, you may need to complete some formatting changes, which you will receive in a follow up email. A member of our team will be in touch with a set of requests shortly. If you do not receive a separate email within a few days, please assume that checks have been completed, and no additional changes are required.

We expect to receive your revised manuscript within two weeks.

To submit your revision, please go to https://www.editorialmanager.com/pbiology/ and log in as an Author. Click the link labelled 'Submissions Needing Revision' to find your submission record. Your revised submission must include the following:

- a cover letter that should detail your responses to any editorial requests, if applicable, and whether changes have been made to the reference list

- a Response to Reviewers file that provides a detailed response to the reviewers' comments (if applicable, if not applicable please do not delete your existing 'Response to Reviewers' file.)

- a track-changes file indicating any changes that you have made to the manuscript.

NOTE: If Supporting Information files are included with your article, note that these are not copyedited and will be published as they are submitted. Please ensure that these files are legible and of high quality (at least 300 dpi) in an easily accessible file format. For this reason, please be aware that any references listed in an SI file will not be indexed. For more information, see our Supporting Information guidelines:

https://journals.plos.org/plosbiology/s/supporting-information

*Published Peer Review History*

Please note that you may have the opportunity to make the peer review history publicly available. The record will include editor decision letters (with reviews) and your responses to reviewer comments. If eligible, we will contact you to opt in or out. Please see here for more details:

https://plos.org/published-peer-review-history/

*Press*

Should you, your institution's press office or the journal office choose to press release your paper, please ensure you have opted out of Early Article Posting on the submission form. We ask that you notify us as soon as possible if you or your institution is planning to press release the article.

*Protocols deposition*

To enhance the reproducibility of your results, we recommend that if applicable you deposit your laboratory protocols in protocols.io, where a protocol can be assigned its own identifier (DOI) such that it can be cited independently in the future. Additionally, PLOS ONE offers an option for publishing peer-reviewed Lab Protocol articles, which describe protocols hosted on protocols.io. Read more information on sharing protocols at https://plos.org/protocols?utm_medium=editorial-email&utm_source=authorletters&utm_campaign=protocols

Please do not hesitate to contact me should you have any questions.

Sincerely,

Ines

--

Ines Alvarez-Garcia, PhD

Senior Editor

PLOS Biology

ialvarez-garcia@plos.org

------------------------------------------------------------------------

DATA POLICY: IMPORTANT - PLEASE READ

You may be aware of the PLOS Data Policy, which requires that all data be made available without restriction: http://journals.plos.org/plosbiology/s/data-availability. For more information, please also see this editorial: http://dx.doi.org/10.1371/journal.pbio.1001797

Note that we do not require all raw data. Rather, we ask that all individual quantitative observations that underlie the data summarized in the figures and results of your paper be made available in one of the following forms:

1) Supplementary files (e.g., excel). Please ensure that all data files are uploaded as 'Supporting Information' and are invariably referred to (in the manuscript, figure legends, and the Description field when uploading your files) using the following format verbatim: S1 Data, S2 Data, etc. Multiple panels of a single or even several figures can be included as multiple sheets in one excel file that is saved using exactly the following convention: S1_Data.xlsx (using an underscore).

2) Deposition in a publicly available repository. Please also provide the accession code or a reviewer link so that we may view your data before publication.

Regardless of the method selected, please ensure that you provide the individual numerical values that underlie the summary data displayed in the following figure panels as they are essential for readers to assess your analysis and to reproduce it:

Fig. 1B-P; Fig. 2A-H, K, L; Fig. 3A-E, G, H, J; Fig. 4A, C, D, F-H, J; Fig. 5A-I; Fig. 6B, D, F, H, J, L; Fig. 7A-C; Fig. S2A-D; Fig. S3A, B; Fig. S4A, B; Fig. S5B; Fig. S6B; Fig. S7B-D and Fig. S8A, B

NOTE: the numerical data provided should include all replicates AND the way in which the plotted mean and errors were derived (it should not present only the mean/average values).

Please also ensure that figure legends in your manuscript include information on WHERE THE UNDERLYING DATA CAN BE FOUND, and ensure your supplemental data file/s has a legend.

Please ensure that your Data Statement in the submission system accurately describes where your data can be found.

***In addition, please make sure that the scRNA-seq raw data files you have deposited in the GEO database are publicly available at this stage (GSE299263).

------------------------------------------------------------------------

Reviewers' comments

Rev. 2:

I commend the authors for their response to reviewer comments. I think the revised manuscript is clearer and more rigorous. Two points which I think could be addressed further:

(1) I'm still unsure about the unit of biological replication in some experiments, which is why I originally suggested using SuperPlots. For instance, Figure 2K aims to show that nod2 mutants have more Lcp1+ cells than wild-type fish. The legend states that each group consists of five fish, yet the graph displays dozens of data points. If a single fish is considered one replicate, shouldn't there only be five data points per group? My earlier SuperPlot suggestion was intended to have the authors add these five fish-level data points to the existing graph, allowing readers to see both the Lcp1+ cells per intestine/ROI and per fish simultaneously. Currently, each dot represents a measurement from a fish intestine image. Is this what counts as a replicate? If so, what is the reasoning, and why not use Lcp1+ cell counts per fish instead? After reviewing the methods section (see below), I still couldn't find clarification. Could the authors explicitly state what constitutes a unit of replication? While variability in results is expected (and reflects underlying biology), choosing different definitions for the unit of replication could impact the conclusions regarding differences between wild type and nod2 mutants.

"Quantification of Anxa4⁺ secretory cells, Lcp1⁺ leukocytes, and TUNEL⁺ apoptotic cells was performed on sagittal intestinal sections using Fiji (ImageJ). Individual villi were manually outlined using the DNA (nuclear) channel to define the epithelial region of interest. Within each villus ROI, Anxa4⁺, Lcp1⁺, and TUNEL⁺ cells were detected using a fixed threshold applied uniformly across all samples and counted manually using the cell counter tool. Total epithelial cell number per villus was determined by counting all DNA⁺ nuclei within the same ROI. To account for differences in villus size and cellular density between sections and fish, data were expressed as the percentage of marker-positive cells per villus, calculated as (number of Anxa4⁺, Lcp1⁺, or TUNEL⁺ cells ÷ total number of DNA⁺ nuclei within the ROI) x 100."

(2) Tamoxifen is a selective estrogen receptor modulator, and I believe the discussion section should more clearly address in the 'limitations' section that the authors are assuming TAM acts solely as an antagonist (though they may have strong reasons for making this assumption). However, TAM can also function as an ER agonist, and it may act as a GPER agonist as well (see PMID 15705806, 16239258). The revised discussion currently states, "…tamoxifen may engage additional target pathways." This wording seems somewhat vague and could be expanded to clarify that there is substantial evidence showing TAM can behave as an agonist in some tissues or contexts and as an antagonist in others, even within the same organism simultaneously, and how this might impact the interpretation of the author's results. This complexity helps explain why TAM cannot be used indefinitely in humans for treating ER-positive breast cancers. That said, I am not insisting on this point and will defer to the editor's judgment regarding how extensively this limitation should be addressed.

# Author comment: Author response to Decision Letter 3

**DOI:** 10.1371/journal.pbio.3003766.r005

31 Mar 2026

Attachment Submitted filename: 26_Response_To_Reviewers_02.pdf

# Editor report: Decision Letter 3

**DOI:** 10.1371/journal.pbio.3003766.r006

**Authors:** Ines Alvarez-Garcia

3 Apr 2026

Dear Dr Foley,

Thank you for the submission of your revised Research Article entitled "Estrogen impacts NOD2-dependent regulation of intestinal homeostasis" for publication in PLOS Biology. On behalf of my colleagues and the Academic Editor, Ken Cadwell, I am delighted to let you know that we can in principle accept your manuscript for publication, provided you address any remaining formatting and reporting issues. These will be detailed in an email you should receive within 2-3 business days from our colleagues in the journal operations team; no action is required from you until then. Please note that we will not be able to formally accept your manuscript and schedule it for publication until you have completed any requested changes.

Please take a minute to log into Editorial Manager at http://www.editorialmanager.com/pbiology/, click the "Update My Information" link at the top of the page, and update your user information to ensure an efficient production process.

PRESS

We frequently collaborate with press offices. If your institution or institutions have a press office, please notify them about your upcoming paper at this point, to enable them to help maximise its impact. If the press office is planning to promote your findings, we would be grateful if they could coordinate with biologypress@plos.org. If you have previously opted in to the early version process, we ask that you notify us immediately of any press plans so that we may opt out on your behalf.

We also ask that you take this opportunity to read our Embargo Policy regarding the discussion, promotion and media coverage of work that is yet to be published by PLOS. As your manuscript is not yet published, it is bound by the conditions of our Embargo Policy. Please be aware that this policy is in place both to ensure that any press coverage of your article is fully substantiated and to provide a direct link between such coverage and the published work. For full details of our Embargo Policy, please visit http://www.plos.org/about/media-inquiries/embargo-policy/.

Many congratulations and thanks again for choosing PLOS Biology for publication and supporting Open Access publishing. We look forward to publishing your study.

Sincerely,

Ines

--

Ines Alvarez-Garcia, PhD

Senior Editor

PLOS Biology

ialvarez-garcia@plos.org
